# Prediction model for patient prognosis in idiopathic pulmonary fibrosis using hybrid radiomics analysis

**DOI:** 10.1016/j.redii.2022.100017

**Published:** 2022-10-29

**Authors:** Daisuke Kawahara, Takeshi Masuda, Riku Nishioka, Masashi Namba, Nobuki Imano, Kakuhiro Yamaguchi, Shinjiro Sakamoto, Yasushi Horimasu, Shintaro Miyamoto, Taku Nakashima, Hiroshi Iwamoto, Shinichiro Ohshimo, Kazunori Fujitaka, Hironobu Hamada, Noboru Hattori, Yasushi Nagata

**Affiliations:** a Department of Radiation Oncology, Graduate School of Biomedical Health Sciences, Hiroshima University, 1-3-2 Kagamiyama, Hiroshima 734-8551, Japan; b Department of Respiratory Medicine, Hiroshima University Hospital, Hiroshima, Japan; c Department of Clinical Oncology, Hiroshima University Hospital, Hiroshima, Japan; d Department Molecular and Internal Medicine, Graduate School of Biomedical Health Sciences, Hiroshima University, Hiroshima, Japan; e Department of Physical Analysis and Therapeutic Sciences, Graduate School of Biomedical and Health Sciences, Hiroshima University, Hiroshima, Japan; f Department of Emergency and Critical Care Medicine, Hiroshima University Hospital, Hiroshima, 734-8551, Japan; g Hiroshima High-Precision Radiotherapy Cancer Center, Hiroshima, Japan

**Keywords:** IPF, Radiomics, Machine learning

## Abstract

**Objectives:**

To develop an imaging prognostic model for idiopathic pulmonary fibrosis (IPF) patients using hybrid auto-segmentation radiomics analysis, and compare the predictive ability between the radiomics analysis and conventional visual score methods.

**Methods:**

Data from 72 IPF patients who had undergone CT were analyzed. In the radiomics analysis, quantitative CT analysis was performed using the semi-auto-segmentation method. In the visual method, the extent of radiologic abnormalities was evaluated and the overall percentage of lung involvement was calculated by averaging values for six lung zones. Using a training cohort of 50 cases, we generated a radiomics model and a visual score model. Subsequently, we investigated the predictive ability of these models in a testing cohort of 22 cases.

**Results:**

Three significant prognostic factors such as contrast, Idn, and cluster shade were selected by LASSO Cox regression analysis. In the visual method, multivariate Cox regression analysis revealed that honeycombing and reticulation were significant prognostic factors. Subsequently, a predictive nomogram for prognosis in IPF patients was established using these factors. In the testing cohort, the c-index of the visual and radiomics nomograms were 0.68 and 0.74, respectively. When dividing the cohort into high-risk and low-risk groups using the median nomogram score, significant differences in overall survival (OS) in the visual and radiomics models were observed (P=0.000 and P=0.0003, respectively).

**Conclusions:**

The prediction model with hybrid radiomics analysis had a better ability to predict OS in IPF patients than that of the visual method.

## 1 Introduction

Idiopathic pulmonary fibrosis (IPF) is a chronic, progressive, and fibrosing interstitial pneumonia of unknown cause, characterized by irreversible destruction of the alveolar structure. The prognosis of IPF is poor, and the median survival is 2–3 years [1], [2], [3]. The clinical course of IPF varies greatly, and while certain cases progress rapidly, some progress slowly [4]. Therefore, the prediction of survival would guide the optimal timing of transplantation for rapidly progressive IPF. Antifibrotic agents approved for clinical use in IPF patients are claimed to slow the disease progression [1], [2], [3] but have significant side effects including gastrointestinal side effects, diarrhea, and liver dysfunction [5]. Thus, the accurate prediction of IPF patients’ prognoses would be useful for selecting appropriate patients who need antifibrotic agents.

IPF prognosis has been evaluated based on forced vital capacity, diffusion capacity for carbon monoxide, and other clinical factors [6], [7], [8]. Ley et al. demonstrated that age, sex, and physiologic score were reliable for predicting the overall survival of IPF patients [8]. Among these, DLco is considered the best parameter reflecting disease severity in IPF patients [9]. However, DLco is inferior to other factors because it has a measurement noise in the range of 5%–15% [10].

IPF shows distinctive features on computed tomography (CT) images. Previous studies showed that the extent of honeycombing, reticulation, and ground-glass attenuation correlated with IPF patients’ prognoses [11, 12]. Chahal et al. predicted the survival of IPF patients based on age, sex, and physiology [13]. These studies visually estimated honeycombing and the percentage of reticulation using high-resolution CT images. However, visual evaluation is limited by the evaluators’ subjectivity. Therefore, computer-aided quantitative evaluation of CT images has been performed to predict lung disease progression. Shin et al. distinguished fibrotic lung from the normal lung using the threshold of CT values [14]. Moreover, Best et al. showed that the kurtosis, skewness, and histogram of CT images correlated with the physiological impairment of this disease [15]. Texture analysis methods have been used to diagnose IPF [16].

IPF is the most common type of interstitial lung disease (ILD), and has a complex morphological pattern and change over time. Flaherty et al. reported that the evaluation and classification of extent are complicated because there is significantly inter- and intra- observer variation [17]. Recently, Lanza et al. showed that the quantitative lung CT analysis that divided the lung region by CT value using a semi-automated segmentation method contributes to predicting the clinical outcome [18].

Radiomics is a specific method that generates high-throughput extraction of a tremendous amount of quantitative imaging data using data-characterization algorithms, and can extract quantitative imaging features that cannot be detected by visual evaluation. Radiomics generally extracts the imaging-based features from the region of interest (ROI) to reflect lesion.

In the current study, we propose hybrid auto-segmentation radiomics analysis as new prediction workflow, which combing the quantitative lung CT analysis and radiomics analysis. It was incorporated into the prognosis prediction model for IPF patients. Moreover, we compared the ability of the prediction between the radiomics model and traditional visual evaluation.

## 2 Materials and methods

### 2.1 Patients and data acquisition

The Review Board of our institution approved this retrospective study (E-939-3). The need for informed consent was waived owing to the retrospective nature of the study. The methods were performed according to relevant guidelines and regulations.

We enrolled IPF patients who were diagnosed at our department between January 2008 and December 2016. IPF was diagnosed according to the American Thoracic Society/Euro-pean Respiratory Society consensus classification 2018, and the method was previously described [11, 19]. Inclusion criteria included patients who underwent CT examination. Exclusion criteria included patients without DICOM.

CT images were acquired during forced inspiration with helical scanning mode, and the field of view included the entire chest. The CT images were obtained using a variety of CT machines, which were analyzed in the previous study [11]. The tube voltage was 120 kV, tube current was 120−400 mA, and slice thickness and slice interval were 5.0 mm. The scanned conventional CT images were used for the radiomics analysis. Lung parenchyma images were used for visual evaluation. CT characteristics were obtained at each patient's first medical examination.

Baseline staging was performed using the gender, age, and physiology (GAP) index: the index was 0−3 for Stage I, 4 and 5 for Stage II, and 6−8 for Stage III.

Follow up was obtained after reviewing initial diagnosis, treatment and cause of death using medical record.

### 2.2 Model

**Fig. 1:**
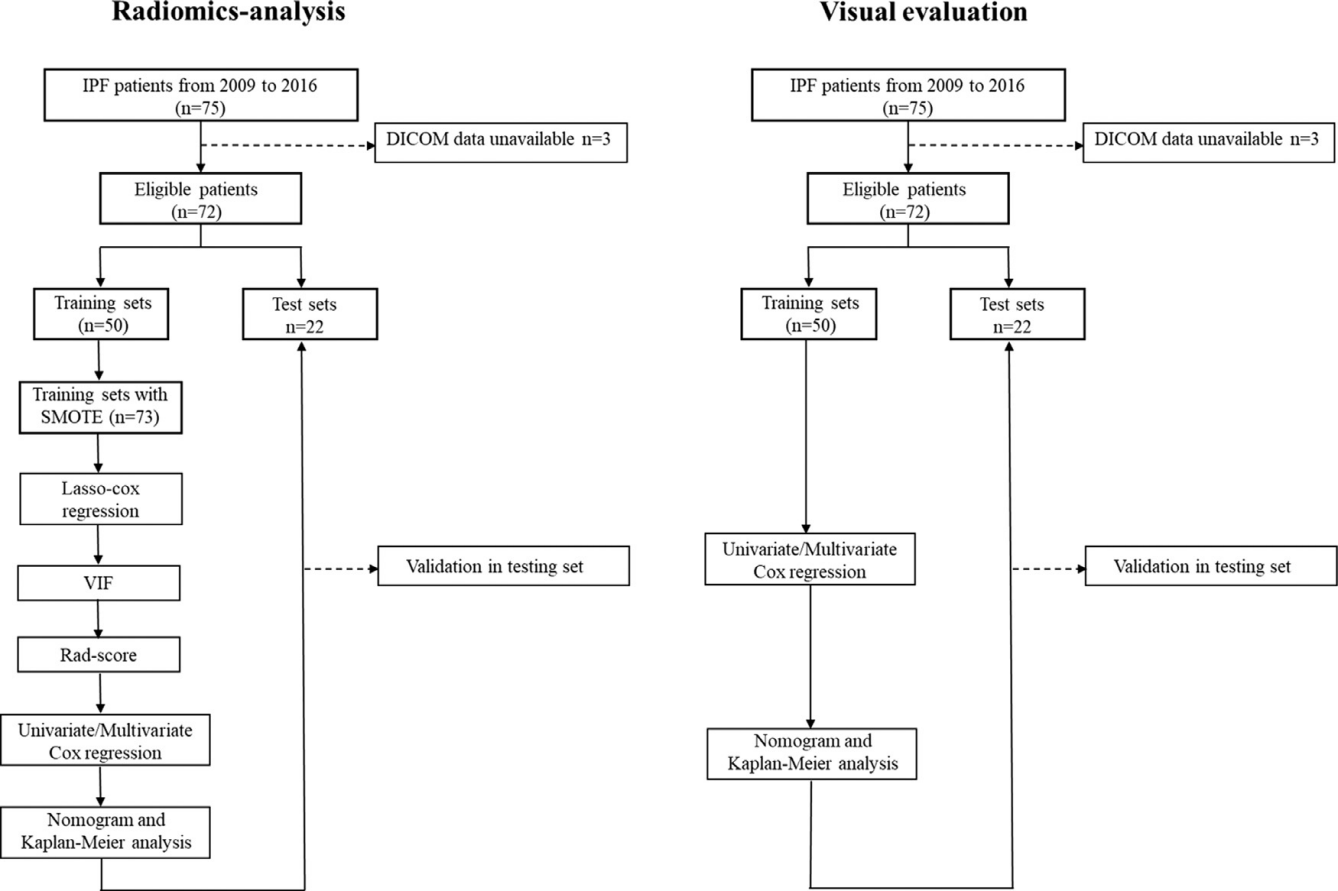
Flowchart of hybrid auto-segmentation radiomics analysis for the prediction of the outcome for IPF patients.

Fig. 1 shows a flowchart of hybrid auto-segmentation radiomics analysis for the prediction of the outcome for IPF patients. First, using a training cohort of 50 cases, we generated a radiomics and a visual score model. Subsequently, we investigated the predictive ability of these models in a testing cohort of 22 cases.

### 2.3 Visual evaluation

The method of visual evaluation of CT findings has been described previously [11]. Briefly, the lungs were divided into six zones, as previously described: 1) left and right upper lung zone, 2) left and right lower lung zone, and 3) left and right middle lung zone, between the upper and lower zones [20]. The extent of lung involvement was evaluated for each of the six lung zones. The score was based on the percentage of the lung parenchyma that showed evidence of abnormalities and was estimated to be the nearest 5% of parenchymal involvement. The overall percentage of lung involvement was calculated by averaging the values for the six lung zones. Moreover, for each lung zone, traction bronchiectasis was scored as 0 (absent) and 1 (present), and all scores were added up.

CT images were independently evaluated by two pulmonologists (pulmonologist 1 with 8 years of experience with IPF CT interpretation and pulmonologist 2 with 6 years of experience with IPF CT interpretation) who were blinded to the clinical data. The discordant results by the two readers were evaluated by pulmonologist 3 with 16 years of experience with IPF CT interpretation, and major opinion was considered the final evaluation of those images. The results of visual evaluation in 75 patients and inter-observer reliability between two readers were previously described [11].

### 2.4 Auto-segmentation

**Fig. 2:**
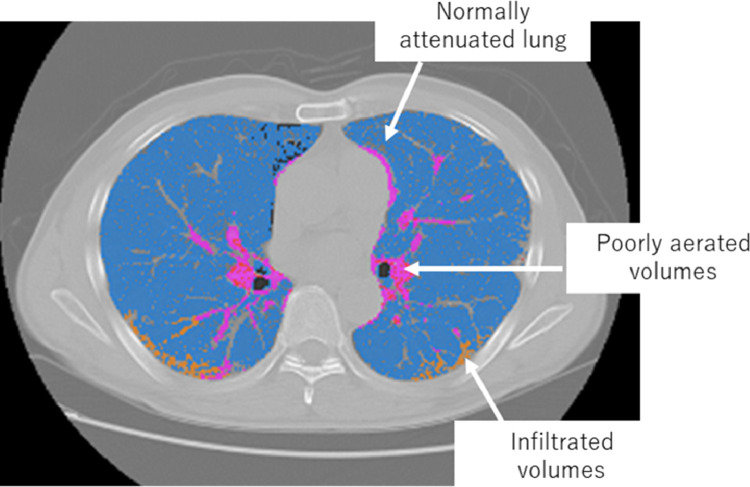
Chest computed tomography (CT) image for IPF patient. The CT was segmented by the threshold CT values. The blue shows normally attenuated patters, the pink shows poorly aerated pattern, and the orange shows infiltrated pattern.

The CT images were transferred to a medical image computing tool (3D Slicer, www.slicer.org) [21] equipped with an automated segmentation algorithm (chest imaging platform) [22]. This software and algorithm were used for quantitative CT analysis of COVID-19. A consistently repeatable analysis of parenchymal impairment can be performed. The whole lung included both lungs with segmentary vessels, interstitial structures, and bronchi. All mediastinal structures were excluded from the bronchi and main pulmonary arteries. Based on the threshold of the CT image, lungs were classified into the normally attenuated lung (−950, −701 HU), infiltrated volumes (−700, −500 HU) and poorly aerated volumes (−500, 100 HU) regions, as shown in Fig. 2.

### 2.5 Radiomics analysis

First, z-score normalization was performed on the whole CT image to transform arbitrary CT values into standardized intensity ranges. Next, radiomics analysis was performed to extract the radiomics features from the auto-segmented region of the CT imaging dataset. Radiomics features were extracted using Pyradiomics, an open-source package in Python [23]. Tables S1 and S2 list the detailed radiomics features. The extracted radiomics features were 21 first-order features, 13 shape features, and 93 texture analysis features. Moreover, the CT images leave the image unchanged as the original image, and these are preprocessed using a wavelet imaging filter. The wavelet filter has a high-pass filter and a low-pass filter. The current study performed eight wavelet decompositions (HLL, LHL, LHH, LLH, HLH, HHH, HHL, and LLL). For example, LHL is interpreted as a high-pass sub-band resulting from the directional filtering of X with a low-pass filter in the x-direction, a high-pass filter in the y-direction, and a low-pass filter in the z-direction. ​A total of 837 features were analyzed for each segmentation in this study.

### 2.6 Subsampling

The synthetic minority over-sampling technique (SMOTE) interpolates data based on the Euclidean distance for variables. In the current study, the 2-year OS was 64.8%. SMOTE can increase the representation of the minority group in the resulting dataset while reflecting the structure of the original dataset [24]. In the current study, SMOTE was used to construct the prediction model on the selected training dataset before least absolute shrinkage and selection operator (LASSO) Cox regression.

### 2.7 Prediction model and statistical analysis

The primary endpoint was the overall survival (OS). Univariate and multivariate Cox regression analyses were performed to identify significant prognostic factors in the visual method. Factors with p-values < 0.05 in the multivariate analysis were selected, and a predictive nomogram for prognosis in IPF patients was established using these factors with the rms package. For the radiomics features, the LASSO Cox regression model is based on the glmnet package, which is suitable for the regression of high-dimensional data. Additionally, interactions between radiomics features were evaluated using variance inflation factor (VIF) analysis. We used the VIF to remove factors with a VIF >10. LASSO Cox regression performs feature selection during model construction by penalizing the respective regression coefficients. As this penalty is increased, more regression coefficients shrink to zero, resulting in a more regularized model. The most significant predictive features were selected from among all the candidate features in the training set with 10-fold cross-validation. Subsequently, the radiomics score was calculated using each patient's selected radiomics features. A predictive nomogram was then established using the rms package. The predictive performance of the nomograms between the visual method and radiomics model was evaluated by the C-index using the Rcorrp.cens package in Hmisc in R. Additionally, we divided the cohort into high-risk and low-risk groups using the median nomogram score. Survival curves were calculated using the Kaplan-Meier method and compared using the log-rank test between the two groups. Statistical significance was set at p < 0.05. The statistical analysis in the current study was performed using the R software package (http://www.r-project.org).

## 3 Results

### 3.1 Patients characteristics and CT score

We enrolled 75 IPF patients, and excluded 3 patients without DICOM data. The clinical characteristics, laboratory data, and pulmonary function tests of the patients are shown in Table 1. The CT scores are also presented in Table 1.

**Table 1:** Clinical Characteristics of training and validation cohort

	Training cohort (n=50)	Validation cohort (n=22)
Patient characteristics		
Observation period (days)	1146.9 ± 806.6	1260.4 ± 903.4
Age	68.3 (46-82)	68.5 (46-85)
Sex (male/female)	43/7	20/2
Brinkman Index	761.7 ± 601.1	737.8 ± 607.2
Blood test		
KL-6 (U/mL)	1348.4 ± 756.5	1245.5 ± 735.6
Pulmonary function test		
FVC (mL)	2490 ± 730 (n=48)	2694 ± 786 (n=20)
FEV_1.0_ (mL)	2053 ± 579 (n=48)	2278 ± 607 (n=21)
DL_CO_ (mL/min/mmHg)	10.4 ± 3.6 (n=46)	12.5 ± 5.1 (n=20)
CT characteristics		
Honeycomb area score (%)	5.15 ± 8.23	6.10 ± 8.68
Reticular area score (%)	7.37 ± 4.78	8.52 ± 5.12
GGA area score (%)	2.68 ± 3.27	5.49 ± 13.1
Consolidation area score (%)	0.20 ± 0.87	0.29 ± 1.39

Follow-up greater than 6 months was available for 65 patients, and mean follow-up duration was 41.6 months +/- (0.5-113.9).

### 3.2 Univariate and multivariate Cox regression analysis for prognostic factors using the visual score

Table 2 shows the results of univariate and multivariate Cox regression analyses for the prognosis of the patient with IPF using visual scores. Univariate analysis revealed that honeycomb, reticulation, and GGA were significant prognostic factors. Further, the multivariate analysis showed that honeycomb and reticulation were also significant prognostic factors for IPF patients.

**Table 2:** Univariate and multivariate Cox regression analysis for the visual scores.

	Univariate analysis		Multivariate analysis	
Factor	HR (95%CI: Low-High)	P-value	HR (95%CI: Low-High)	P-value
honeycomb	1.03 (1.00-1.06)	0.031	1.03 (1.00-1.06)	0.046
reticulation	1.09 (1.04-1.15)	< 0.001	1.07 (1.01-1.14)	0.03
consolidation	1.22 (0.94-1.15)	0.13	-	-
GGA	1.06 (1.02-1.10)	0.001	1.03 (0.981.09)	0.21

Abbreviations: CI = confidence interval; HR=hazard ratio;GGA=ground-glass attenuation

### 3.3 Selected features by LASSO Cox regression in the radiomics analysis

Table 3 lists the selected radiomics features using LASSO Cox regression. A total of 3348 features were extracted from CT images in the radiomics analysis. We extracted radiomics features such as contrast, idn and clustershade. Contrast, Idn were selected from the entire lung region and clustershade was selected from poorly aerated regions. All features used a wavelet filter. Although radiomics analysis was performed in the normal attenuated lung, poorly aerated lung, infiltrated lung, and entire lung regions, the radiomics feature as a predictor was not selected from the normally attenuated lung and infiltrated volumes were not selected by LASSO Cox regression.

**Table 3:** Selected features by Lasso-Cox regression for radiomics analysis.

Region	Filter	Item	Feature-type
Whole lung	Wavelet-LLH	Ngtdm	Contrast
Whole lung	Wavelet-LLH	Glcm	Idn
Poorly aerated	Wavelet-HHH	Glcm	Clustershade

### 3.4 Nomograms for predicting the 1-year and 2-year survival using the visual and radiomics models

The nomograms for predicting the 1-year and 2-year survival using the visual model and radiomics model are presented in Fig. 3A, Fig. 3B, respectively. For each IPF patient, the nomogram assigned points based on the visual score and radiomics score (Rad-score). The total point was calculated by summing the individual points. The nomogram graphically calculates the scale, point, and total point. For example, in the visual score model, the honeycomb score; 20 was converted to 32.5 points and reticular score; 10 was converted to 17 points. Finally, we added 32.5 and 17 points to calculate the total points. Patients with a lower Rad-score had a better OS. The C-index of the visual score model and the Rad-score model were 0.68 and 0.74 in the testing cohort, respectively.

**Fig. 3A:**
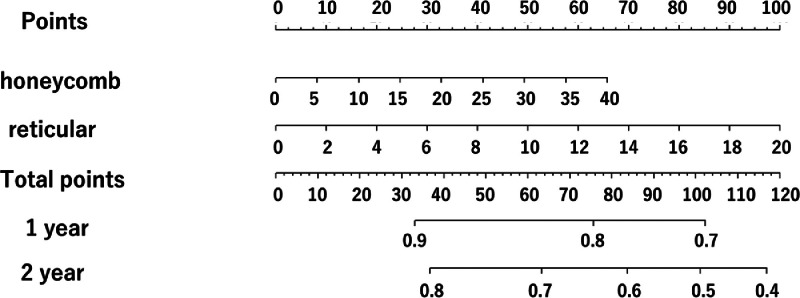
Nomogram for prediction of 1-year and 2-year survival with the visual score model.

**Fig. 3B:**
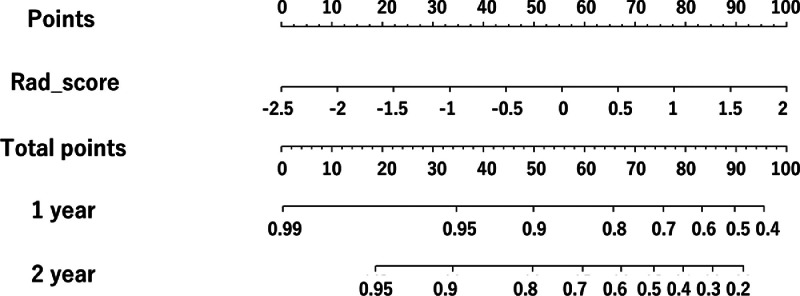
Nomogram for prediction of 1-year and 2-year survival with the radiomics model.

### 3.5 Survival and prediction of 1-year and 2-year survival with the visual model and radiomics model

The OS was compared between the high-risk and low-risk groups in the visual model or radiomics model using Kaplan-Meier analysis, as shown in Fig. 4A, Fig. 4B. The performance of the radiomics signature was confirmed in the testing cohort, and a significant difference was observed between the high-risk and low-risk groups in both the 1-year and 2-year OS rates (P < 0.001). The low-risk groups with the Rad-score model had a better OS. To compare the predictive power of GAP model, which is validated clinical prediction model and radiomics model, we compared the prognosis between IPF patients with GAP score 1 and 2-3 or GAP score 1-2 and 3. As shown in Fig. 4C, Fig. 4D, there were no significant differences between two groups.

**Fig. 4A:**
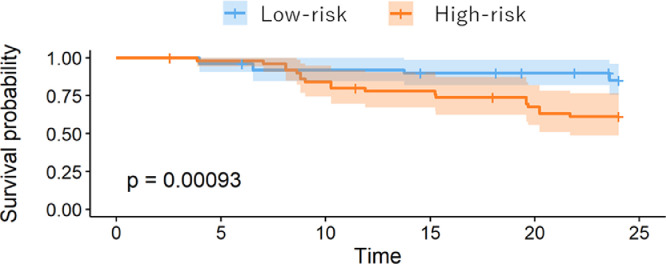
Kaplan-Meier survival curves for overall survival stratified by median nomogram score using the visual score model.

**Fig. 4B:**
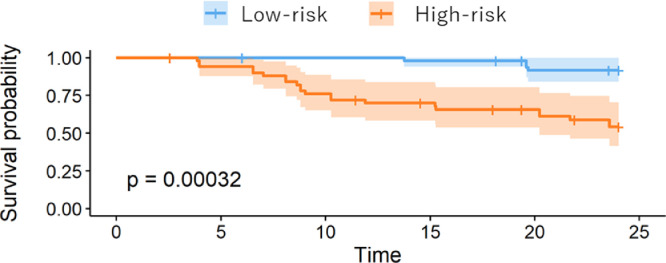
Kaplan-Meier survival curves for overall survival stratified by median nomogram score using radiomics model.

**Fig. 4C:**
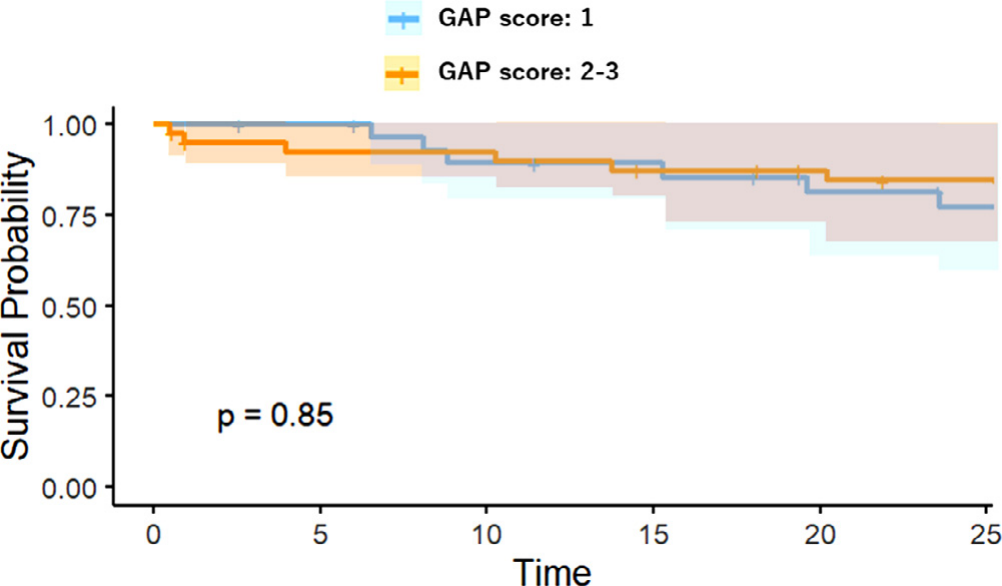
Kaplan-Meier survival curves for overall survival stratified by the GAP score 1 and 2-3.

**Fig. 4D:**
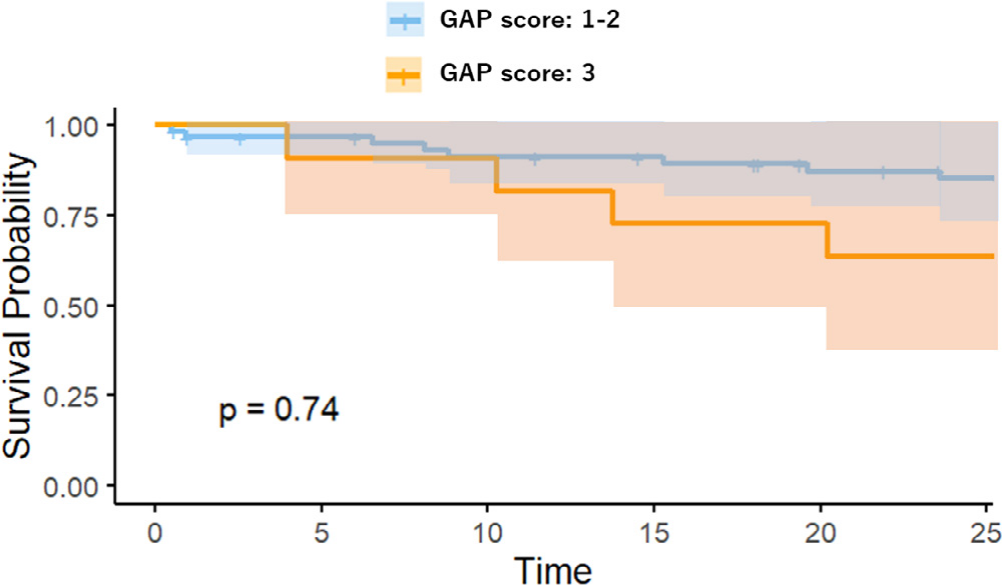
Kaplan-Meier survival curves for overall survival stratified by the GAP score 1-2 and 3.

## 4 Discussion

A prediction of the individual overall survival contributes to guiding the optimal timing of transplantation and making appropriate treatment decisions for IPF patients. The current study developed a hybrid auto-segmentation radiomics model for predicting prognosis in IPF patients, and showed that this radiomics model was superior to a conventional visual evaluation.

Conventionally, the extent of CT findings by visual evaluation has been used to predict OS in IPF patients [11]. Lynch et al. reported that the presence of reticulation and honeycomb is an important independent predictor of OS in IPF patients [25]. The current study also showed that nomogram-based prediction using visual scores of reticulation and honeycomb were significant prognostic factors for IPF patients. However, the visual score depends on the evaluators’ experience and expertise. That is, it is both subjective and qualitative.

Radiomics can extract quantitative imaging features that cannot be detected by visual evaluation. Previously, we proposed a multi-region radiomics analysis that can extract features in the local and whole lung regions [26]. Although it improved the accuracy of the prediction of radiation pneumonitis after radiotherapy, the segmentation was manually performed by the physician. Moreover, Shin et al. showed that a simple method with thresholds of CT values could distinguish lung function [14]. The current study developed a hybrid auto-segmentation radiomics analysis, which extracted the radiomics features from the focused region, such as the poorly aerated or infiltrated region that is segmented automatically. We classified the lungs into three regions quantitatively based on threshold CT numbers. Moreover, we extracted 3348 radiomics features from segmented and whole lung regions. The significant radiomics features for the prediction of OS in IPF patients were selected in the whole lung and poorly aerated lung regions. The nomogram results showed that patients with lower contrast, Idn, and clustershade on the CT image had a better prognosis.

Our radiomics analysis showed that higher contrast, Idn, and clustershade were significant poor prognostic factors. These values represent lower uniformity and higher skewness in the CT image. Lower uniformity and higher skewness indicate the presence of reticular and honeycomb structures in the lung. In a previous study, Park et al. also showed that texture features could quantify reticular and honeycombing [27]. From these observations, we consider that our radiomics analysis quantifies the extracted signature, which has been regarded as important of the IPF in the visual score on the CT image. In the current study, we showed that radiomics analysis was superior to visual evaluation. In fact, the C-index for the Rad-score model was higher than that of the visual score model. This indicates that the hybrid radiomics analysis method has a better prediction performance of the OS for IPF patients than visual evaluation. This finding is the reason why hybrid radiomics analysis does not only quantify the signature, but also extracts the predictor of survival for patients with IPF that cannot be detected in the visual score. On the other hand, the total volume of intestinal abnormalities and volume of reticular can be a predictor of the mortality [28]. Although the current study evaluated the volume of intestinal abnormalities in the normal attenuated lung, poorly aerated lung, infiltrated lung, and entire lung regions, we may not be able to accurately detect the volume. Bartoli et al. proposed a deep learning segmentation model of COVID-19 lung lesions and diffuse lung disease [29, 30]. Further study will be performed to develop the auto segmentation method for the IPF and improve the accuracy of the OS prediction.

The current study has limitations.

First, it used CT images obtained from variety CT scanners in a single institution.

Second, in the prognosis prediction with the radiomics research, the dataset is generally divided into training/validation/test, and an external testing dataset is usually a better option to evaluate the real diagnostic performances. The current study used a limited CT images obtained from a single institution. Therefore, we generated a radiomics model using a training cohort, and we investigated the predictive ability of these models in a testing cohort alone. A versatile prediction model will be created by including multiple institutions and other IPF patients. Third, observational period was less than 6 months in 12% of testing cohort or 4.5% of validation cohort. Observational period was not enough to investigate prognosis in patients with IPF.

The current study showed that a prediction model with hybrid radiomics analysis using semi-auto chest CT segmentation demonstrated a better ability to predict OS in IPF patients compared to a regular visual method.

## Disclosure of potential conflicts of interest

None

## Author contributions

DK and TM analyzed data and wrote the main manuscript text. MN, NI, RN, KY, SS, YH, SM, TN, HI, SO, KF, HH, NH, and YN commented on the manuscript and the research plan.

## Ethnical approval

All procedures performed in studies involving human participants were in accordance with the ethical standards of the institutional and/or national research committee and with the 1964 Helsinki declaration and its later amendments or comparable ethical standards.

## Informed consent

The Review Board of our institution approved this retrospective study (E-939-3). The need for informed consent was waived owing to the retrospective nature of the study. The methods were performed according to relevant guidelines and regulations.

## Funding

None

## Declaration of Competing Interest

None
